# Editorial: Highlights in Heart Failure and Transplantation in 2021

**DOI:** 10.3389/fcvm.2022.877515

**Published:** 2022-03-25

**Authors:** Matteo Cameli, Emma J. Birks, Federico Landra

**Affiliations:** ^1^Division of Cardiology, Department of Medical Biotechnologies, University of Siena, Siena, Italy; ^2^Division of Cardiovascular Medicine, University of Louisville, Louisville, KY, United States; ^3^Division of Cardiovascular Medicine, University of Kentucky, Lexington, KY, United States

**Keywords:** heart failure, radiation, drugs, bioimpedance, seismocardiogram, machine learning, rehabilitation

## Space Travels to Elucidate Radiation-Induced CV Disease

More than 50 years after Neil Armstrong first set foot on the moon, space travel is gaining new popularity. However futuristic this topic may seem, humankind will probably undertake space travel on a broader scale in the upcoming centuries or even decades. This means that people will need to learn how to cope with challenges related to microgravity, hypoxia, disrupted circadian rhythms, and radiation exposure. The evidence raised from radiation exposure for curative aims and nuclear disasters has led the scientific community to recognize and understand its multiple pathogenic effects on the human body's various systems, including cardiovascular one. Meerman et al. carefully examined the issue of radiation exposure related to long-distance space travel, with a particular focus on its cardiovascular effects. The pathogenic mechanisms that can lead to radiation-induced cardiovascular disease (RI-CVD), including myocardial remodeling and fibrosis, atherosclerosis, and microvascular damage, are elucidated. In addition, potential countermeasures and protection methods are reported, from physical shielding to pharmacological means.

## New Technologies Ready for Clinical Use

The future is entering our lives not only by enhanced horizons but also with new technologies. Bioimpedance spectroscopy (BIS) is a non-invasive method that relies on the different electrical impedance of biological tissues to assess fluid volume status. A clinical trial by Accardi et al. showed that BIS-measured extracellular fluid was higher in patients with heart failure compared to healthy individuals and that this result was consistent with the echocardiographic parameters of fluid status, such as inferior vena cava size. It was suggested that this tool may contribute to the risk stratification of patients with heart failure and facilitate clinical decision-making both in the clinic and potentially at home. Another tool of increasing study is seismocardiography (SCG), which allows the non-invasive estimation of stroke volume, cardiac output, and myocardial contractility through cardiac and blood-induced motions transmitted to the chest surface as vibratory phenomena. In an original research article, Morra et al. show that SCG can quantify cardiac kinetic energy and continuously track its changes during an acute myocardial infarction and subsequent reperfusion. As it has already happened for atrial fibrillation with smartwatches, thanks to its ease of use SCG could potentially help in the follow-up of heart failure patients monitoring their myocardial mechanical activity.

## Machine Learning for Disease Course Prediction

Above all the technological advances that are presented to us nowadays, the one that is playing an increasingly prominent role is with no doubt machine learning. Machine learning is a branch of artificial intelligence that uses statistical methods to enhance the performance of an algorithm to identify patterns in data. Fahmy et al. used machine learning for predicting heart failure progression in a huge cohort of patients affected by hypertrophic cardiomyopathy (HCM). Heart failure progression was defined as worsening in NYHA class, a drop in left ventricular ejection fraction, need for septal reduction procedure, and/or indication for heart transplantation. A set of 17 clinical and imaging variables, also confirmed by an independent validation dataset, were identified as the most important predictors of progressive heart failure in HCM patients.

## Advances in Heart Failure Understanding and Treatment

Besides new technologies, an important branch of research remains the one whose aim is to identify biological molecules with a potential clinical application for diagnostic and prognostic purposes. This is the case with Big Endothelin-1, the prepropeptide of endothelin-1 (ET-1), the most potent endogenous vasoconstrictor, which has been recently recognized as an independent predictor of short-term adverse events in acute decompensated heart failure by Mo et al. The predictive value of big ET-1 was comparable to NT-proBNP; moreover, the combined use of the two molecules increased the predictivity of the primary outcome, defined as a composite of in-hospital death, cardiac arrest, and utilization of mechanical circulatory support. Many articles also take into account chronic heart failure and much interest addresses the more ambiguous categories, namely heart failure with mildly reduced (HFmrEF) and preserved ejection fraction (HFpEF). Regarding the latter, Abramov and Parwani criticized the tendency to gather all patients with preserved ejection fraction under a unique definition, since this group of patients is very heterogeneous. These authors suggest not focusing on the cutoffs identified by the recently introduced diagnostic score algorithms (HFA-PEFF and H_2_FPEF) but instead trying to understand the underlying pathophysiology to come to better management decisions. The other equivocal category is HFmrEF. Patients can fall into this subgroup even though they may have different backgrounds. The diction “mildly reduced” implies that the ejection fraction used to be normal before. However, since ejection fraction is a dynamic state, an improvement is also possible, which underlies the emerging concept of recovered/improved ejection fraction. Therefore, HFmrEF could be “mildly reduced” as much as “mildly recovered” ejection fraction. Zhang et al. demonstrated that HFmrEF patients with previously preserved ejection fraction have worse outcomes compared to those with a previously reduced ejection fraction. Definitions of heart failure are continuously evolving as well as therapeutic options. Pascual-Figal et al. have undertaken a comprehensive review on Sacubitril-Valsartan, a game-changer drug not only in the field of HFrEF.

## Prevention and Rehabilitation: Never Too Early, Never Too Late

Drugs are not the only way of managing heart failure. Huang et al. undertook an interesting systematic review on the effects of Tai Chi exercise among adults with chronic heart failure, which shows potential as a cardiac rehabilitation discipline thanks to its low-intensity, making it suitable for people with poor exercise tolerance. Finally, diametrically opposite to rehabilitation and of equal importance, there is prevention for people with cardiovascular risk factors. It is a common opinion that prevention and rehabilitation will cover more and more space in the following decades to ensure a better quality of life and at the same time improve health care sustainability. Among the most common cardiovascular risk factors, there is diabetes, with more than 500 million people affected worldwide. Chadalavada et al. analyzed an enormous UK dataset and found that mortality and heart failure risk were almost doubled in people with diabetes compared to those without it, this being more evident for female patients.

## Future Directions

We get better and better at identifying risk factors for diseases' development and progression, at predicting their course, and deploying increasingly effective therapies at the right time. For the whole spectrum of time frames in heart failure course, we know what to do in order to either prevent it or improve the prognosis and quality of life of our patients. Research continuously casts light on previously unsolved dilemmas in clinical management.

## Author Contributions

All authors listed have made a substantial, direct, and intellectual contribution to the work and approved it for publication.

## Conflict of Interest

The authors declare that the research was conducted in the absence of any commercial or financial relationships that could be construed as a potential conflict of interest.

## Publisher's Note

All claims expressed in this article are solely those of the authors and do not necessarily represent those of their affiliated organizations, or those of the publisher, the editors and the reviewers. Any product that may be evaluated in this article, or claim that may be made by its manufacturer, is not guaranteed or endorsed by the publisher.

